# New insights into the generation and role of de novo mutations in health and disease

**DOI:** 10.1186/s13059-016-1110-1

**Published:** 2016-11-28

**Authors:** Rocio Acuna-Hidalgo, Joris A. Veltman, Alexander Hoischen

**Affiliations:** 1 Department of Human Genetics, Radboud Institute for Molecular Life Sciences, Radboud University Medical Center, Geert Grooteplein 10, 6525 GA Nijmegen, The Netherlands; 2 Department of Human Genetics, Donders Institute of Neuroscience, Radboud University Medical Center, Geert Grooteplein 10, 6525 GA Nijmegen, The Netherlands; 3 Department of Clinical Genetics, GROW – School for Oncology and Developmental Biology, Maastricht University Medical Centre, Universiteitssingel 50, 6229 ER Maastricht, The Netherlands

## Abstract

Aside from inheriting half of the genome of each of our parents, we are born with a small number of novel mutations that occurred during gametogenesis and postzygotically. Recent genome and exome sequencing studies of parent–offspring trios have provided the first insights into the number and distribution of these de novo mutations in health and disease, pointing to risk factors that increase their number in the offspring. De novo mutations have been shown to be a major cause of severe early-onset genetic disorders such as intellectual disability, autism spectrum disorder, and other developmental diseases. In fact, the occurrence of novel mutations in each generation explains why these reproductively lethal disorders continue to occur in our population. Recent studies have also shown that de novo mutations are predominantly of paternal origin and that their number increases with advanced paternal age. Here, we review the recent literature on de novo mutations, covering their detection, biological characterization, and medical impact.

## Introduction

Upon fertilization, a human zygote inherits half of its genome from the mother via the oocyte and the other half from the father through the sperm. In addition to the genetic information passed on from generation to generation, each of us is born with a small number of novel genetic changes—de novo mutations—that occurred either during the formation of the gametes or postzygotically [1, 2]. Additionally, novel mutations continue arising throughout post-natal and adult life in both somatic and germ cells. Only mutations present in the germ cells can be transmitted to the next generation [3].

**Fig. 1:**
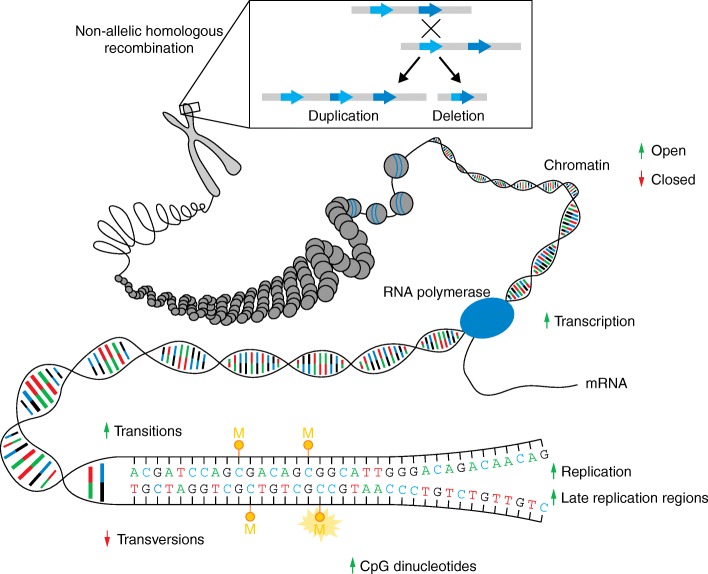
Mechanisms of de novo mutations. De novo mutations can arise because of static properties of the genome, such as the underlying sequence (deamination of methylated CpGs, transitions versus transversions) or due to erroneous pairing of nucleotides during DNA replication. However, de novo mutations can also occur in relation to cell-specific properties such as the chromatin state, transcriptional status, and gene expression levels. Mutational hotspots for genomic rearrangements are largely determined by the underlying genomic architecture. One such example is given for non-allelic homologous recombination (NAHR). *Arrows* represent the influence of each feature on the de novo mutation rate. *Green arrows pointing upwards* indicate elevated mutability; *red arrows pointing downwards* indicate lower mutability. *M* methyl group modifying cytosine

There is a long-standing interest in the study of the frequency and characteristics of de novo mutations in humans, as these are crucial to the evolution of our species and play an important role in disease. A typical human genome varies at 4.1 to 5.0 million positions compared with the human reference genome [4]. The vast majority of genetic variation observed in a typical human genome is common and shared by more than 0.5% of the population as a result of having been recombined, selected, and passed on for many generations [4]. By contrast, a typical human genome contains 40,000 to 200,000 rare variants that are observed in less than 0.5% of the population [4]. All of this genetic variation must have occurred as a de novo germline mutation in an individual at least once in human evolution [5]. Historically, the germline mutation rate in humans has been calculated by analyzing the incidence of genetic disorders; in 1935, Haldane estimated the mutation rate per locus per generation based on the prevalence of hemophilia in the population [6, 7]. More recently, in 2002, Kondrashov accurately calculated the de novo mutation rate in humans by examining the mutation rate at known disease-causing loci [8]. Nowadays, next-generation sequencing (NGS) approaches in parent–offspring trios can be used to directly study the occurrence of all types of de novo mutations throughout the genome, from single-nucleotide variants (SNVs) to small insertions–deletions (indels) and larger structural variations (Box 1). Genome-wide NGS studies place the germline de novo mutation rate for SNVs in humans at 1.0 to 1.8 × 10^–8^ per nucleotide per generation [1, 9–13], with substantial variation among families [11, 13, 14]. This number translates into 44 to 82 de novo single-nucleotide mutations in the genome of the average individual, with one to two affecting the coding sequence [9, 10, 12, 13, 15]. These state-of-the art genomic approaches allow us to determine additional characteristics of de novo mutations, such as the parental origin and whether they occurred in the germline or postzygotically. We now know that the majority of germline de novo mutations have a paternal origin and that a higher paternal age at conception results in an increase in the number of de novo mutations in the offspring [15–18]. Furthermore, the study of large cohorts of parent–offspring trios provides insight into the distribution of mutations throughout the genome, the genomic context in which they arise, and possible underlying mechanisms [11–13] (see Fig. 1 for an overview of different mechanisms resulting in de novo mutations).

Mutations conferring a phenotypic advantage propagate rapidly through a population [19–21], whereas neutral mutations can disseminate merely as a result of genetic drift [22]. However, damaging mutations resulting in deleterious traits before or during the reproductive phase undergo purifying selection, and their spread through the population is averted [23]. This entails that de novo mutations are genetically distinct from inherited variants, as they represent the result of the mutagenic processes taking place between one generation and the next, before undergoing selection (Table 1). Loss or acquisition of traits at the population level drives evolution of a species, whereas, at the level of an individual, loss or acquisition of traits can result in disease.

**Table 1:** Comparison of inherited and de novo variants

	Inherited variants	De novo mutations
Single-nucleotide variants (SNVs)	3.5 to 4.4 million [4]	44 to 82 [9, 10, 12, 13, 15]
Number of coding SNVs	22,186 [10]	1–2 [25]
Insertions and deletions (indels <50 bp)	~550,000 [4]	2.9–9 [26, 91]
Large indels (50–5000 bp)^a^	~1000 [4]	0.16 [26]
Copy-number variations (CNVs)	~160 [4]	0.0154 [26]^b^
Selection pressure in previous generation(s)	High	None
Damaging capacity of variants	Majority with small effect	High
Differences in population	Yes	None
Parental/paternal age effect	None	Strong
Detection of variants	Imputable	Not imputable
Amenable to positional cloning^c^	Yes	No

^a^Owing to technical limitations, the number and mutation rate for large indels ranging between 50 and 5000 bp remain uncertain. Novel sequencing approaches will likely provide more-accurate estimates (see Chaisson et al. [205])

^b^Per generation for CNVs larger than 100 kb

^c^Positional cloning by linkage analysis or homozygosity mapping

Germline de novo genetic alterations have been implicated in human disease for decades. Virtually all disease-causing aneuploidies arise as de novo events. The best known example for this is trisomy 21, identified in 1959 as the cause of Down syndrome [24]. In the beginning of this millennium, genomic microarray technology provided insight into the role of de novo copy-number variations (CNVs) in disease [25]. Even though large CNVs occur at a very low rate, arising at a frequency of only 0.01 to 0.02 events per generation [25–27], they contribute significantly to severe and early-onset neurodevelopmental disorders and congenital malformations owing to their disruptive effect on many genes [28]. The magnitude of the contribution of de novo genetic alterations to human disease, however, has only recently become fully apparent now that NGS approaches allow the reliable and affordable detection of all types of de novo mutations [25]. Damaging de novo point mutations and indels affecting important genes in development have been established as a prominent cause of both rare and common genetic disorders [29–35].

In this review, we first touch on the biological aspects of de novo mutations in humans, such as their origin, distribution throughout the genome, and factors related to their occurrence and timing. Later, we discuss the increasingly recognized role of de novo mutations in human disease and other translational aspects. Throughout, we will focus mostly on de novo SNVs; readers should refer to Box 2 and previous work from others for more information on the role of de novo CNVs and other structural genomic variation in human disease [36, 37].

## Causes of de novo mutations

Mistakes during DNA replication can give rise to de novo mutations as a result of the erroneous incorporation of nucleotides by DNA polymerases [38]. DNA polymerases ε and δ catalyze replication predominantly in the leading and lagging strand, respectively. Both polymerases integrate nucleotides during polymerization in a highly selective way, with an average of one mismatch per 10^4^–10^5^ bp in vitro [39, 40]. A proofreading subunit present in both polymerases subsequently verifies the geometry of the paired nucleotides to ensure that the incorporated base is correct [38].

Single or multiple base-pair mismatches can cause alterations in the structure of the replicating DNA and can be restored by the mismatch repair (MMR) pathway [41]. The MMR pathway is highly efficient, which explains why the amount of mutations generated during DNA replication is much lower than the polymerase error rate. The frequency at which specific base-pair substitutions arise can be different from the speed at which they are repaired, which defines the mutation rates for specific base-pair substitutions [41]. Incomplete repair can lead to single or multiple base-pair substitutions or indels. Additionally, damaged nucleotides can be incorporated during replication, leading to mispairings and base substitutions [42].

DNA lesions can also appear spontaneously as a consequence of exogenous or endogenous mutagens—UV or ionizing radiation and DNA-reactive chemicals are an example of the former, whereas reactive oxygen species belong to the latter [38]. Before replication, these spontaneous lesions are repaired mainly by the nucleotide excision repair system and base excision repair pathways [43]. However, inefficient repair of pre-mutations before a new round of DNA replication can lead to the mutation becoming permanently fixed in either one or both daughter cells [44]. If mutation repair fails, DNA replication might also be completely arrested and ultimately lead to cell death [44].

The difference between the rate at which pre-mutagenic damage appears in DNA and the rate at which it is repaired defines the rate at which de novo mutations arise. It is often assumed that germline de novo mutations originate from errors in DNA replication during gametogenesis, particularly in sperm cells and their precursors (see section below on parental origin of de novo mutations). However, inefficient repair of spontaneous DNA lesions can also give rise to de novo mutations during spermatogenesis, as continuous proliferation and short periods between cell divisions can translate into there being less time to repair these lesions [44, 45]. Furthermore, in oogenesis, spontaneous DNA mutations coupled to inefficient repair mechanisms might play a more prominent role [44]. Therefore, while the de novo mutation rate is a reflection of the replication error rate and the number of mitoses a cell has undergone, this number is also influenced by the amount of time between mitoses and the efficiency of the DNA repair [44].

## Distribution of de novo mutations in the genome

While the typical human mutation rate is 1–1.8 × 10^–8^ per nucleotide per generation [1, 9–13], mutagenesis does not occur completely at random across the genome [9]. Variation in mutability across different areas of the genome can be explained by intrinsic characteristics of the genomic region itself, related to its sequence composition and functional context [46]. Certain factors playing a role in the mutability of the genomic region are predicted to be shared by all cell types in the human organism. These include the local base-pair context, recombination rate, and replication timing [9, 13, 47]. Replication timing refers to the order in which different areas of the genome are replicated during the S-phase of the cell cycle. Genomic regions that are replicated late display more genetic variation than regions that are replicated early [47]. It has been suggested that this could be due to a higher mutability that is secondary to depletion of dNTPs at the end of replication, although other changes such as alterations in polymerase activity and decreased MMR repair activity have also been implicated [38, 48, 49].

Other factors influencing mutability can vary from cell to cell, depending on the transcriptional activity and chromatin state [50–52]. In addition, recent whole-genome sequencing (WGS) studies have revealed the presence of so-called “mutational clusters” and “mutational hotspots”. Mutational clusters correspond to the observation of multiple de novo mutations in very close vicinity in a single individual, whereas multiple de novo mutations occurring at the same location in several individuals are an indication of the existence of mutational hotspots [53].

### Nucleotide differences: transitions, transversions, and CpGs

The molecular events underlying transitions occur more frequently than those leading to transversions, resulting in a two-fold greater rate of transitions over transversions across the genome [27, 38]. Transitions arise predominantly as a result of C > T mutations, which is at least partially explained by the mutability of CpG dinucleotides [54]. The cytosine in a CpG dinucleotide often undergoes methylation at the fifth position of the six-atom ring, leading to 5-methylcytosine (5-mC). In humans, methylated CpG dinucleotides are known to be chemically unstable and highly mutable due to deamination of 5-mC at CpG dinucleotides, resulting in G:T mismatches [12]. Indeed, the mutability of CpG dinucleotides is approximately ten to eighteen times higher than that of other dinucleotides [27], and, as a result, CpG dinucleotides are found at only a fraction of their expected frequency in the human genome [54]. The high de novo mutation rate at CpG sites is also illustrated by the recent work of the Exome Aggregation Consortium (ExAC). Through the work of this consortium, exome data from more than 60,000 individuals without severe pediatric disease are currently available (Box 3). Analysis of the data in ExAC shows that the discovery of new mutations at CpG dinucleotides reaches saturation at 20,000 exomes [55, 56]. This emphasizes that identical CpG mutations do not necessarily reflect an ancestral event but are likely the result of independent de novo mutations.

Remarkably, the mutability of CpG dinucleotides is lower in genomic regions enriched for CpG and with higher GC content than in the rest of the genome [44]. In fact, the mutation rate for CpGs in the GC-richest regions of the genome are two to threefold lower than in the rest of the genome [44, 48]. This could be the result of lower methylation levels, the effect of selection because the regions play a role in gene regulation, or secondary to stronger binding between DNA strands impeding separation and spontaneous deamination [38, 44, 57].

### Mutational signatures underlying specific mutational processes

While errors in DNA replication, exposure to mutagens, or failure to repair DNA damage can all result in mutations, there are differences in the pattern of mutations arising from each of these processes. A “mutational signature” has been defined as a pattern of mutations that is specific to a mutational process occurring in a cell, tissue, or organism [58]. A recent study based on the analysis of 4.9 million somatic mutations in more than 12,000 cancer genomes defined 21 mutational signatures associated with mutational processes active in somatic cells (termed signature 1 to 21) [58]. Detailed descriptions of each signature are available at http://cancer.sanger.ac.uk/cosmic/signatures. Each of these millions of mutations is placed into one of 96 possible mutation types based on six possible base pair substitutions (C > A, C > G, C > T, T > A, T > C, and T > G) and one of four possible base pairs adjacent to the mutation both at the 5′ and at the 3′ position of the mutation. Concisely, each mutation type is a trinucleotide in which the middle base pair is mutated to a specific nucleotide and each mutational signature is defined by the frequency of each mutation type observed [59].

A recent study showed that the mutational spectrum of germline de novo mutations correlated best with two of these previously described mutational signatures, currently known as signatures 1 and 5 [11, 13]. This suggests that the mutational processes associated with these signatures in somatic cells might also be active in germ cells, although the mechanisms underlying the processes remain elusive. Mutational signature 1 represents close to 25% of de novo germline mutations and is characterized by a high proportion of C > T transitions at CpG dinucleotides, which is associated with deamination of methylated cytosine [11, 58]. Mutational signature 5, which corresponds to the remaining 75% of de novo mutations, is characterized mainly by A > G transitions [11]. While the mechanism underlying this signature remains unclear, the mutations observed as part of this signature might be secondary to spontaneous deamination of adenine to hypoxanthine, which is then read as guanine [60]. This mutational signature is associated with transcriptional strand bias, suggesting that some of these mutations arise from adducts subject to transcription-coupled repair [60].

### Mutational clusters and hotspots

De novo mutations occur throughout the human genome, but occasionally several mutations can arise at a closer distance than expected by random distribution [9]. The term “mutational clusters” refers to the occurrence of de novo mutations in an individual at a closer distance than expected, with multiple de novo mutations within regions ranging from 10 to 100 kb [9, 12, 13, 53]. Mutational clusters display a unique mutational spectrum, with a lower rate of transitions and a large proportion of C > G transversions [13]. This phenomenon has been described to arise in somatic cells in the context of cancer, where it is known as “kataegis”, and is linked to the family of enzymes known as APOBEC (for “apolipoprotein B mRNA editing enzyme, catalytic polypeptide-like”) [53, 58]. It has been suggested that clusters involving C > G transversions could be related to the formation of single-stranded DNA in diverse cellular processes, such as double-strand breaks and dysfunctional replication forks [61]. Single-stranded DNA might be mistaken for retroelements and attacked by APOBEC enzymes, which convert cytosine to uracil [53]. The mutations are then repaired through base-excision repair and subsequent translesional DNA synthesis with error-prone polymerases [38]. Indeed, mutational clusters have been described to be reminiscent of APOBEC-mediated mutations, albeit with a different sequence context [12, 13]. The occurrence of mutational clusters has been found to correlate with increased parental age [13].

Another origin for some of these clusters could be chromosomal rearrangements. It has been shown that the mutation rate for SNVs is elevated and SNVs can cluster in proximity to the breakpoints of de novo CNVs [62, 63]. This is likely the result of the replicative CNV mechanism in which a low-fidelity, error-prone DNA polymerase is used during repair of DNA. Indeed, work performed in yeast supports the observation that double-strand-break-induced replication is a source of mutation clusters [61].

In contrast to the mutation clusters that occur within one individual, mutational hotspots are considered overlapping loci that are found to be mutated more frequently than expected in different individuals. Recent research based on WGS datasets and modeling has identified such hotspots in coding sequences [9]. Furthermore, the existence of these mutational hotspots has been recently confirmed in a larger study that showed specific bins of 1 Mb within the human genome with elevated mutation rates [13]. Interestingly, in this study, two bins including genes *CSMD1* and *WWOX* were shown to have a higher maternal than paternal mutation rate. The mechanism for this is still largely unknown, but the latter is a well-known fragile site within the human genome [64]. Other sites of the human genome that are especially prone to de novo mutations include ribosomal DNA (rDNA) gene clusters [65], segmental duplications [66], and microsatellites [67], with mutation rates three to four orders of magnitude higher than average [68].

## Parental origin of de novo germline mutations

In human embryos, the primordial germ cells (PGCs) emerge from the epiblast, eight to fourteen cell divisions after fertilization [69]. In these first cell divisions, the mutation rate appears to be similar in male and female embryos (approximately 0.2–0.6 mutations per haploid genome per cell division, according to models estimating the mutation rate during gametogenesis) [11]. After their specification, PGCs expand to form the pool of spermatogonial stem cells and the complete population of primary oocytes in male and female embryos, respectively [11, 69]. Despite differences in the expansion of PGCs to oogonia or spermatogonia, the mutation rate during this step is similar in both sexes, with approximately 0.5 to 0.7 mutations per haploid genome per cell division, according to computational modeling [11]. However, after puberty, the processes involved in spermatogenesis and oogenesis diverge further. Spermatogonial stem cells divide by mitosis approximately every 16 days, maintaining the spermatogonial stem cell pool while generating differentiated spermatogonial cells which produce sperm cells through an additional round of mitosis followed by meiosis [70]. By contrast, each menstrual cycle, a few oocytes escape from meiotic arrest and complete the first meiotic division. After ovulation, the oocyte becomes arrested once more until fertilization, when it completes the second meiotic division. Thus, after PGC expansion in embryogenesis, oocytes only undergo one additional round of DNA replication in their evolution to a mature ovum. In contrast, spermatogonial cells can undergo hundreds of rounds of DNA replication and cell division before their maturation to sperm cells.

**Fig. 2:**
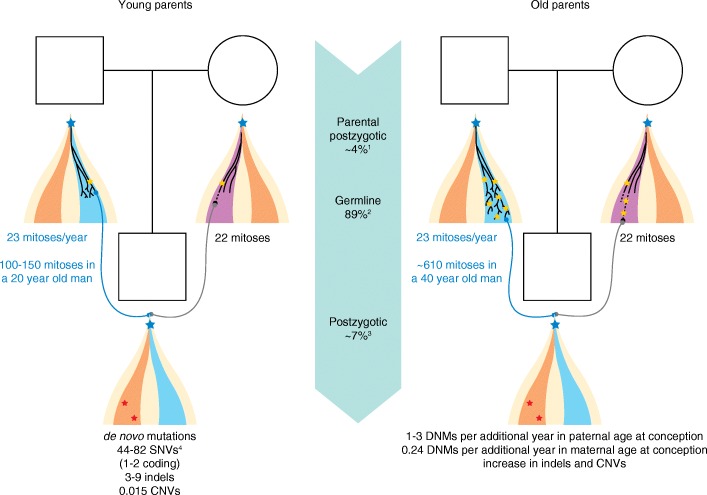
Timing of de novo mutations (DNMs). Sperm cells have undergone approximately 100 to 150 mitoses in a 20-year-old man, whereas oocytes have gone through 22 mitoses in a woman of the same age (*left*). As a result of errors in both replication of the genome and repair of DNA damage occurring during parental embryogenesis, gametogenesis, or as postzygotic events in the offspring, DNMs arise in each new generation. Advanced parental age is associated with an increase in the number of de novo mutations (*right*). The male germline adds 23 mitoses per year, entailing that a spermatogonial stem cell in a 40-year-old man has undergone more than 600 cell mitoses. Each additional year in paternal age at conception adds one to three de novo mutations to the genome of the offspring. Oogenesis has a fixed number of mitoses, but mutations accumulate over time possibly owing to failure to repair DNA damage. The increase in number of de novo mutations with maternal age is lower: 0.24 extra de novo mutations for each additional year of maternal age at conception. Cell lineages modified from [238]. Somatic cells are showed in *orange*, the male germline is shown in *blue*, and the female germline is shown in *purple. Blue stars* represent postzygotic mutations present in the germline and in somatic cells; *yellow stars* represent mutations arising exclusively in the germline; *red stars* represent somatic mutations arising during embryonic development or post-natal life which are absent from germline cells. Figure footnotes: ^1^The ratio of paternal to maternal mutations originating from parental gonosomal mosaicism is 1:1; ^2^the ratio of paternal to maternal germline de novo mutations is 4:1; ^3^the ratio of paternal to maternal postzygotic de novo mutations is 1:1; ^4^this range is based on the average number of de novo mutations published elsewhere [9, 10, 12, 13, 15] irrespective of parental age

Approximately 80% of all de novo germline point mutations arise on the paternal allele, and advanced paternal age at conception has been established as the major factor linked to the increase in the number of de novo mutations in the offspring, both at the population level and within the same family (Fig. 2) [11, 13, 15]. Spermatogonial cells continue to divide throughout life, which is likely to allow the progressive accumulation of mutations due to errors during DNA replication but also as a result of failure to repair non-replicative DNA damage between cell divisions [44]. Furthermore, the efficiency of endogenous defense systems against radical oxygen species and of DNA repair mechanisms might also decline with age [71, 72]. De novo mutations in children of young fathers show a different signature and localize to later-replicating regions of the genome compared with those of children of old fathers, suggesting that additional factors contribute to de novo mutations with age [12, 13]. It has been calculated that one to three de novo mutations are added to the germline mutational load of the offspring for each paternal year at conception, but this effect varies considerably between families [11, 13]. This variability has been suggested to be due to individual differences in the rate of mutagenesis, in the frequency of spermatogonial stem cell division and even to genetic variation in DNA mismatch repair genes [11]. Indeed, one could speculate that deleterious variation in genes involved in replication and repair could predispose to elevated de novo mutation rates not only in somatic cells but also in the germline, as has been observed in mouse models lacking exonuclease activity in DNA polymerase δ [73].

The effect of increased maternal age is well established for errors leading to chromosomal nondisjunction involved in aneuploidies [74, 75], but less so for de novo point mutations. The fixed number of mitoses required for oogenesis would entail that maternal age would not be linked to an increase in DNA-replication-associated mutations. However, an effect of maternal age on the number of de novo mutations has been reported recently [13, 76], likely reflecting an excess of non-replicative DNA damage that is not properly repaired [44]. This maternal age effect was initially reported in a study analyzing de novo mutations in WGS data from a large cohort of parent–offspring trios, in which maternal age correlated with the total number of de novo mutations after correcting for paternal age [76]. A more detailed analysis of the same cohort confirmed a subtle but significant increase in the number of maternal de novo mutations with advancing maternal age, comprising 0.24 additional de novo mutations per extra year of maternal age at conception [13]. Previous studies had failed to identify a maternal age effect on the number of de novo mutations [12, 15]. This might be explained by differences in the parental age distribution between cohorts or due to a lack of statistical power to detect this subtle effect for which paternal age is a confounder [76]. The increase of de novo mutations with advanced paternal and maternal age support the possibility that the accuracy of DNA repair mechanisms in germ cells decreases with age [72].

### Selective advantage of de novo mutations in the testes

A striking increase with paternal age has been observed for a small subset of de novo mutations that are highly recurrent and localize to specific nucleotides in the genome. These de novo mutations are thought to grant spermatogonial stem cells a growth advantage, leading to clonal expansion of mutated cells in the testis [77]. For instance, gain-of-function mutations in genes in the RAS–MAPK pathway have been shown to cause clonal expansion of mutant spermatogonial stem cells owing to proliferative selective advantage [77, 78]. Computational modeling suggests that this would result from a slightly increased ratio of symmetric versus asymmetric divisions in mutant spermatogonial stem cells, favoring the production of two mutated spermatogonial stem cells compared with a single mutated stem cell and one differentiated spermatogonial stem cell harboring the mutation [79, 80]. Therefore, over time, spermatogonial stem cells carrying these mutations undergo positive selection owing to higher self-renewal than surrounding wild-type cells and expand clonally in the testis [81]. The occurrence and enrichment of mutations in spermatogonial stem cells is thought to take place in all men and would entail that the testes of older men contain a higher number of clones of mutant spermatogonial stem cells [77, 78].

Interestingly, the first mutations implicated in clonal expansion in spermatogonial stem cells were initially shown to cause developmental disorders such as Noonan and Costello syndrome (caused by *PTPN11* and *HRAS* mutations, respectively) [78, 81, 82], Apert, Crouzon, and Pfeiffer syndromes (*FGFR2*) [81, 83], achondroplasia, Muenke syndrome and thanatophoric dysplasia (*FGFR3*) [81, 82], and multiple endocrine neoplasia (*RET*) [84]. Mutations that are positively selected at the spermatogonial stem cell level but are detrimental at the organism level have been termed to behave selfishly and are therefore referred to as “selfish mutations” [82]. Owing to the expansion of mutant cells over time, the incidence of these developmental disorders shows an exponential increase with paternal age at conception, well beyond the increase observed for other disorders caused by de novo mutations [85]. Appropriately, these disorders are known as “recurrent, autosomal dominant, male-biased, and paternal” (RAMP) age effect disorders or, simply, paternal age effect (PAE) disorders [45, 78]. Because of the selfish selection of mutant spermatogonial cells, PAE disorders have an incidence up to 1000-fold higher than expected based on the mutational target size and the average mutation rate [45, 85]. It has been hypothesized that “selfish mutations” with a weaker effect on spermatogonial stem cell behavior could be involved in more-common phenotypes, such as intellectual disability, autism, or epilepsy [86]. Furthermore, “selfish” behavior is a characteristic of certain mutations driving cancer as they lead to positive cellular selection despite being harmful for the organism. Predictably, several mutations behaving selfishly in spermatogonial stem cells have also been identified as somatic events driving clonal growth in tumorigenesis [82].

Following the identification of genomic regions enriched for maternal de novo mutations [13], the possibility of selfish mutations in the maternal germ line has also been put forward [72]. It appears that these genomic regions harbor genes with a role in tumor suppression, and some de novo mutations could, it is speculated, provide mutant oocytes in aging women with a survival advantage over wild-type ones [72].

## Timing of de novo mutations

De novo mutations have traditionally been considered to occur as germline events, but the advent of NGS allowed scientists to demonstrate that de novo mutations occur as non-germline events more often than previously estimated [3, 87–89]. Mosaicism, which is the existence of two or more genetically distinct cell populations in an individual developing from a single fertilized egg [90], is the norm rather than the exception. Postzygotic mutations, that is, mutations arising in the first few cell divisions after fertilization, can lead to high-level mosaicism and be present in many different tissues of an organism. Mutations that arise later in development or post-natal life, by contrast, can remain restricted to a single tissue or even to a small number of somatic cells (Fig. 2).

Approximately 7% of seemingly de novo mutations are present in blood as high-level mosaic mutations, having likely occurred as early postzygotic events [88, 89, 91]. This, together with the observation that chromosomal instability and structural rearrangements are common in cleavage-stage human embryos, has led to the suggestion that early embryogenesis might be a period of high mutability [92, 93]. Before the initiation of transcription and translation in the zygote, human embryos rely on maternal proteins contributed by the oocyte [94], which could lead to a shortage of proteins involved in DNA replication and repair, resulting in genomic instability [3]. Depending on the timing at which a de novo mutation arises during embryonic development, it could be present at different levels in multiple tissues or be organ specific [95]. A recent study examined multiple samples from the same individual and showed the widespread presence of postzygotic de novo mutations in tissues of different embryonic origin, including somatic and germ cells [96]. Furthermore, mutations can arise in the germ cell lineage after the specification of PGCs during early embryonic development, remaining isolated from somatic cells [3]. Although these mutations are undetectable in sampled tissues such as blood or buccal swabs, they can be transmitted to the offspring as germline events.

Somatic cells are predicted to accumulate hundreds of different mutations throughout post-natal and adult life [97]. Large chromosomal abnormalities have been observed in many tissues in the human body [98], such as the blood, where the presence of these lesions increases with age [99–101]. For instance, loss of the Y chromosome in blood cells has been described as a frequent event in aging males, affecting over 15% of men aged 70 years or older [102, 103]. Somatic mutations resulting in low-level mosaicism are prevalent in healthy tissues [104], including the brain [105], blood [106–108], and skin, where the somatic mutation rate has been calculated at two to six SNVs per megabase of coding sequence per cell [109]. As a result of the accumulation of somatic mutations, the genome sequence is certain to vary among different cells of an individual, a level of genetic diversity that is best observed with single-cell sequencing technologies [110]. Studies in mouse models have shown that the mutation frequency is higher in somatic cells than in germ cells [111, 112]. The comparison of the somatic and germline mutation rate in humans supports this finding, which might stem from differences in the efficiency of DNA replication and repair mechanisms in germ and somatic cells, in addition to differences in exposure to mutagens [72].

## De novo mutations in human disease

The medical relevance of de novo mutations has only recently been fully appreciated, mainly because advances in sequencing technology have allowed a comprehensive analysis of these mutations [25]. The field of human genetics had previously focused primarily on inherited diseases, leaving sporadic disorders largely untouched. This was because traditional disease gene identification methods relied mainly on positional mapping of disease loci in large pedigrees with multiple affected members, followed by Sanger sequencing to identify disease-causing mutations in candidate genes. By contrast, NGS techniques such as whole-exome sequencing (WES) or WGS now provide the possibility to detect most, if not all, genetic variation present in a patient. To this end, trio-based WES or WGS has been instrumental in detecting and characterizing de novo mutations in patients with a wide variety of diseases (Box 1) [25, 35].

### De novo mutations in pediatric disease

De novo mutations are now well known to play an important role in severe early-onset diseases, which for the most part arise sporadically because of their impact on fitness; owing to the severity of the phenotype in which they often result, an individual with a deleterious de novo mutation will not produce offspring and the phenotype therefore only arises through de novo mutations.

In the first 5 years of widespread availability of WES, more than 500 novel disease–gene associations have been identified, with the strongest increase in sporadic diseases caused by de novo mutations [35, 113, 114]. Recent studies applying exome sequencing in the clinic have shown that of all sporadic cases that received a molecular diagnosis through clinical exome sequencing, between 60 and 75% could be explained by de novo mutations [115, 116]. De novo mutations affecting the coding region have also been established as an important cause of common neurodevelopmental disorders, such as autism [29, 30], epilepsy [31], and intellectual disability [33, 34], which affect over 1% of the population [117, 118]. Clearly, these common genetic disorders are not explained by de novo mutations affecting the same locus in every patient. Instead, an extreme genetic heterogeneity is observed, and patients with common genetic disorders carry de novo mutations in many different genes. The population frequency of a disorder caused by de novo mutations is determined in large part by the number of genes or genetic loci that can result in this disorder when mutated, which we have referred to previously as the “mutational target” [25]. Rare disorders are most often caused by mutations in a single gene or a small number of genes, while common genetic disorders usually have a large mutational target, often comprising hundreds to thousands of genes or genetic loci. [25]. As an example, more than 700 genes have now been identified to cause autosomal dominant intellectual disability when mutated [117], and this number is rapidly increasing since the widespread application of NGS technology. Based on these sequencing studies, it appears that the majority of the most severe neurodevelopmental phenotypes, such as severe intellectual disability with an IQ below 50, are the consequence of damaging de novo germline mutations in the coding region [10]. An enrichment for damaging de novo mutations has also been observed in individuals with milder phenotypes such as autism spectrum disorder without cognitive deficits [16, 18, 29, 30, 119]. For these milder phenotypes that have less impact on fitness, the exact contribution of de novo mutations to the disease burden is not yet firmly established, and inherited variation is likely to be at least as important in the expression of the phenotype [120–122]. Next to neurodevelopmental disorders, de novo mutations also play a prominent role in pediatric diseases such as congenital heart defects (CHDs) [123–125]. In agreement with the observation made in neurodevelopmental disorders, recent studies found the highest contribution of de novo mutations to disease in individuals with the most severe and syndromic forms of CHD [123, 125]. Finally, it is essential in large-scale sequencing studies to test formally whether the recurrence of de novo mutations in a gene exceeds the number of observations expected by chance (Box 3) [126].

The vast majority of pathogenic de novo mutations are involved in dominant genetic disorders. This appears logical, as a single damaging de novo mutation can be sufficient to cause these kinds of disorders. However, there are examples of recessive disorders that can be caused by the combination of an inherited mutation on one allele and the occurrence of a de novo mutation on the other [33]. In a cohort of 100 trios with severe ID, we identified one case of autosomal recessive ID that was due to the inheritance of one pathogenic allele and the occurrence of a de novo hit in the other [33], and similar observations in the context of late-onset disease are described below. Furthermore, there are reports of cases with a merged phenotype comprising two clinically distinct disorders of which either one or both are caused by a pathogenic de novo mutation [115]. Phenotype-based and classic genetic approaches are insufficient to diagnose individuals with this kind of combined disease, illustrating the power of an unbiased genotype-first approach. Additionally, this approach reduces the need for clinical homogeneity for disease–gene identification studies, as was required for phenotype-first approaches [127, 128].

### De novo mutations in late-onset disorders

Few studies until now have addressed the role of de novo mutations in late-onset diseases. The role of de novo mutations is likely to be smaller in late-onset disorders than in pediatric disorders given the effect of de novo mutations on reproductive fitness. Nevertheless, genes involved in adult-onset disorders are just as likely to be affected by de novo mutations as genes involved in pediatric disorders. A complicating factor in these late-onset disorders, however, is the collection of parental samples for the study of de novo mutations [129]. Despite this obstacle, recent publications have suggested a link between de novo mutations and late-onset neurological and psychiatric disorders: Parkinson’s disease, amyotrophic lateral sclerosis, schizophrenia, and bipolar disorder have been associated with de novo SNVs and CNVs [130–137]. For example, one study found that 10% of individuals with sporadic schizophrenia have a rare de novo CNV compared with 1.26% for controls [132]. Exome sequencing of a cohort of 623 schizophrenia trios identified an enrichment for de novo point mutations in genes encoding synaptic proteins in cases compared with controls [130]. A large meta-analysis recently identified both an excess of loss-of-function mutations in the histone methyltransferase *SETD1A* and an excess of de novo occurrence of these mutations in individuals with schizophrenia compared with controls [138]. Recent studies have exposed a genetic overlap between neurodevelopmental disorders and schizophrenia, with de novo mutations in the same gene being involved in both early and late-onset disorders [138–140]. While de novo mutations have been firmly linked to neurodevelopmental disorders, their involvement in late-onset psychiatric phenotypes is more controversial. This could be the result of a more complex underlying genetic architecture [141], together with a more prominent role for environmental factors in the expression of the phenotype [142].

Cancer, particularly in relatively young individuals without relevant family history, has been associated with de novo mutations in genes involved in cancer-predisposition syndromes. For example, at least 7% of germline mutations in *TP53* (encoding cellular tumor antigen p53) in individuals with Li-Fraumeni syndrome occurred de novo [143], and a similar proportion has been identified for mutations in *APC* involved in familial adenomatous polyposis [144]. Nevertheless, the rate of de novo mutations in genes involved in other cancer-predisposition syndromes, such as *BRCA1* and *BRCA2* [145], or in DNA mismatch repair genes (*MLH1*, *MSH2*, *MSH6*, and *PMS2*) [146] has been reported to be much lower.

Interestingly, de novo mutations have also been identified as causative mutations in genetic disorders that are typically inherited, such as hereditary blindness. For instance, the rate of causative de novo mutations among sporadic cases within a cohort of patients with retinitis pigmentosa was close to 10% [147], a result that was later confirmed by an independent study [148]. Although for the majority of this group the de novo mutation represented a single dominant hit causative of the phenotype, in one case the de novo mutation was in fact the second hit in an autosomal recessive form of retinitis pigmentosa. Similarly, in a cohort suffering from mild-to-moderate sensorineural hearing loss, de novo mutations were identified in two out of eleven sporadic cases [149], also suggesting a role for de novo mutations in this heterogeneous disorder.

As de novo mutations are known to play an important role in disorders that affect fitness, it might also be very relevant to investigate their role in disorders linked to fertility, such as male infertility. Both de novo chromosome Y deletions as well as de novo point mutations in a few genes have been found to cause this disorder [150, 151], but a systematic screen is lacking so far.

### Postzygotic de novo mutations in disease

The timing of a pathogenic de novo mutation can have an important influence on the expression of the phenotype. Postzygotic mutations are currently receiving more and more attention as technological improvements allow the detection of (low level) mosaic mutations for the first time at a genome-wide scale (Box 1). Postzygotic de novo mutations have been identified as the cause of several human diseases, ranging from developmental disorders [152–154] to cancer [155–157]. While de novo mutations arising later in development and leading to gonadal or gonosomal mosaicism might be clinically silent in that individual, there is an increased likelihood that the mutation is transmitted to the offspring as a germline event, resulting in a clinical disorder [158].

Regardless of whether they occur in the germline or postzygotically, some de novo mutations lead to a single Mendelian phenotype in which the mosaic and constitutive form are part of the same clinical spectrum [159]. For example, pathogenic mutations in genes involved in epileptic encephalopathies [160] and cerebral cortical malformations [161] have been shown to cause similar phenotypes when they arise either in the germline or as postzygotic de novo mutations leading to mosaicism in the brain. However, in some of these cases, mosaicism might cause a clinical phenotype milder than a constitutive mutation [162, 163].

De novo mutations can also result in different phenotypes when they are present in the germline or arise postzygotically [164]. Some de novo mutations lead to developmental disorders only if the de novo mutation occurs postzygotically, as the constitutive presence of the mutation is suspected to be lethal [165, 166]. Examples of this include Proteus syndrome (caused by *AKT1* mutations) [152], Sturge-Weber syndrome (*GNAQ*) [153], and CLOVES syndrome (*PIK3CA*) [167]. A common feature to these disorders is that they are caused by mutations known to lead to activation of cellular proliferation pathways and overgrowth. The mutations with the strongest effect generally result in more-severe developmental alterations [168], suggesting that the type of de novo mutation influences the expression of the phenotype. Remarkably, the mutations with the strongest effect on activation have also been observed as somatic events in cancer [168], for which constitutive activation of cellular proliferation pathways is a major hallmark [169]. This finding supports the view that not only the type of pathogenic mutation but also the time at which the mutation occurs is crucial in defining its consequences.

The timing of a postzygotic mutation determines the percentage of affected cells in the organism and the type of tissues involved [90, 153]. For instance, the same genetic alteration in genes in the RAS–MAPK pathway can result in very diverse phenotypes, depending on the timing at which they arise [164, 170, 171]. Mutations in *HRAS* mutating codon G12 of the HRAS protein have been identified in Costello syndrome when present in the germline [172], but postzygotic and embryonic occurrences of mutations in this residue have been observed in Schimmelpenning syndrome [164], sebaceous nevus [164], keratinocytic epidermal nevi [173], and early-onset bladder cancer [157, 174]. Furthermore, identical mutations in the phosphoinositide-3-kinase *PIK3CA* can cause different phenotypes, ranging from different overgrowth syndromes [154] to lymphatic [175] and venous malformations [176], depending on the tissue distribution. Therefore, the timing of a pathogenic de novo mutation is likely instrumental in defining its phenotypic consequences as it determines the burden placed by the mutation upon the organism, including the type of tissues affected and the percentage of cells in which the mutation is present [90, 153].

Finally, an important characteristic of postzygotic mutations is that they generate genetically distinct populations of cells that coevolve within a single organism. This can lead to competition between populations of cells [177] or generate interference in signal transduction between cells [178, 179]. For example, craniofrontonasal syndrome is an X-linked disorder in which women with germline mutations and men with postzygotic mutations have a more severe phenotype than men with germline mutations, owing to interference in cell signaling between different cell populations [179].

Postzygotic de novo mutations have been implicated in early-onset cancer [155, 157] and could well represent an early mutational event in the development of cancer in the general population [156]. Additionally, the high degree of mosaicism observed in a normal human brain has led to the suggestion that pathogenic postzygotic and somatic mutations could be at the source of psychiatric disorders [180, 181]. The role of mosaic de novo mutations is not yet fully appreciated, and it is to be expected that our understanding of this class of mutations will increase rapidly in the coming years because of further technological improvements as well as access to DNA from other (affected) tissues or even cell-free DNA (cfDNA) as a source of DNA from multiple tissues [182–184].

## De novo mutations in clinical practice

The recent recognition of the importance of de novo mutations in human disease has many implications for routine genetic testing and clinical practice. De novo mutations are now established as the cause of disease in a large fraction of patients with severe early-onset disorders, ranging from rare congenital malformation syndromes [185, 186] to more-common neurodevelopmental disorders, such as severe forms of intellectual disability [33], epilepsy [31], and autism [29]. Together, these disorders represent a substantial proportion of all patients seen at neuropediatric and clinical genetics departments around the world.

Pinpointing the genetic cause of a disorder caused by a de novo mutation in an individual can be challenging from the clinical point of view because of pleiotropy as well as genetic heterogeneity underlying a single phenotype. For instance, intellectual disability can be caused by de novo point mutations, indels, or CNVs in any of hundreds of genes [117]. This obstacle to providing a clinical diagnosis strongly argues for a reliable and affordable genomics approach that can be used to detect these de novo mutations in large groups of patients. Exome and genome sequencing (which additionally offers the possibility of accurate detection of structural variation) of patient–parent trios is ideal for this and will soon become the first-tier diagnostic approach for these disorders. A key advantage of this trio-based sequencing approach is that it helps prioritize candidates by de novo occurrence, allowing clinical laboratories to focus on the most likely candidate mutations for follow-up and interpretation (Box 3) [187]. The interpretation of candidate de novo mutations can be guided by the use of different scores, such as the “residual variation intolerance score” (RVIS), based on the comparison of rare versus common missense human variation per gene [188]. Alternatively, “selective constraint scores” can be used, based on the observed versus expected rare functional variation per gene within humans [126].

The identification of a de novo mutation as the cause of disease in a patient has several implications for the patient and his or her family. First, the detection of the genetic defect underlying the phenotype establishes a genetic diagnosis that can be used to provide a prognosis based on data from other patients with similar mutations [189] and information about current treatment options [190] and, in the future, for the development and application of personalized therapeutic interventions [191]. Furthermore, the identification of a de novo mutation offers the parents of the affected patient an explanation as to why the disorder occurred and might help deal with feelings of guilt [192, 193]. In terms of family planning, the identification of a de novo mutation as the cause of disease in a child can be positive news with regard to recurrence risk, as it is much lower than for recessive or dominant inherited disorders (slightly above 1% versus 25 and 50%, respectively) [11, 158]. However, the recurrence risk is strongly dependent on the timing of the mutation as parental mosaicism for the mutation increases the risk of recurrence [158]. Approximately 4% of seemingly de novo mutations originate from parental mosaicism detectable in blood [11], and recent work suggests that transmission of parental mosaicism could explain up to 10% of de novo mutations in autism spectrum disorder [194]. This entails that a fraction of de novo mutations have an estimated recurrence risk above 5% [158]. Furthermore, close to 7% of seemingly de novo mutations arise as postzygotic events in the offspring [88, 89, 91]. Parents of an individual with a postzygotic mutation have a low risk for recurrence of the mutation in an additional child, estimated as being the same as the population risk [90]. Targeted deep sequencing of a disease-causing mutation can be performed to test for its presence in parental blood and detect mosaicism in the offspring. Although it is not yet offered on a routine basis, this kind of testing can provide a personalized and stratified estimate of the recurrence risk based on the presence or absence of mosaicism in the parents or in the offspring.

Finally, it is impossible to prevent de novo mutations from arising in the germline of each new generation, but attention must be brought to the factors that increase the number of de novo mutations in the offspring. The single most important risk factor is advanced paternal age at conception [15], which is of great importance from an epidemiological perspective since most couples in Western countries are having children at later ages. In fact, this increase in de novo mutations with paternal age at conception might explain epidemiological studies that link increased paternal age to increased risk of neurodevelopmental disorders in offspring [195]. A recent population-genetic modeling study, however, indicated that de novo mutations might not explain much of the increased risk of psychiatric disorders in children born to older fathers [122]. While this might be the case for relatively mild and later-onset phenotypes such as schizophrenia, de novo mutations are responsible for the majority of the most severe pediatric disorders arising in outbred populations [10, 196]. At present, most attention, advice, and guidelines are focused on advanced maternal age as a public health issue. It is evident from current work on de novo mutations that advising the public, including policy makers, on potential risks of advanced paternal age and the burden it might bring on society is crucial. An extreme “solution” if reproduction is to be postponed might be to promote cryopreservation of oocytes and sperm [197], a measure under much debate that has been termed “social freezing”.

## Conclusions and future directions

Advances in sequencing technologies have provided us with the ability to identify systematically most if not all de novo mutations in a genome. This has boosted fundamental research into the evolution of our genome by providing insight into the mechanisms that play a role in mutagenesis, the origins of these mutations, and their distribution throughout the genome. While most of this research has been focused on germline mutations, we now see a shift towards the detection and study of somatic de novo mutations also for non-cancer phenotypes, greatly facilitated by more accurate and deeper-coverage sequencing technologies. Next-generation sequencing has also boosted research and diagnostics on sporadic diseases. The routine detection of de novo mutations by trio-based sequencing of patients and their unaffected parents in research as well as in diagnostics will soon allow the identification of most disease-causing genes involved in sporadic monogenic disorders. This will allow for the classification of different developmental and neurodevelopmental disorders based on the underlying genotype rather than solely on the phenotype. In turn, this offers the possibility of targeted medical consultations and interventions, engagement in gene-specific patient groups, and, in some cases, treatment. The study of de novo mutations will shift more and more towards the detection and characterization of non-coding de novo mutations in disease. Although a phenomenal challenge that will require large-study cohorts and detailed functional validation, the limited number of de novo mutations per genome reduces the search space for pathogenic non-coding mutations, as was shown recently for non-coding de novo CNVs [198].

## Box 1 Sequencing technology and de novo mutations

Whole-exome sequencing (WES) and whole-genome sequencing (WGS) provide the possibility to perform an untargeted exome- or genome-wide analysis of an individual’s DNA and, in theory, detect all the genetic variation present in an individual. By applying these approaches in parent–offspring trios, one can determine which variants are inherited and which have occurred as de novo mutations in the offspring. The trio design shown in Box Fig. 1a allows investigators to focus directly on the 44 to 82 de novo mutations arising in the human genome per generation. Most current technologies rely on re-sequencing, which is short-read sequencing followed by mapping and comparison to the human reference genome [199], relying on the raw sequencing quality [200] as well as the mapping quality of the NGS reads [201].

**Box Fig. 1:**
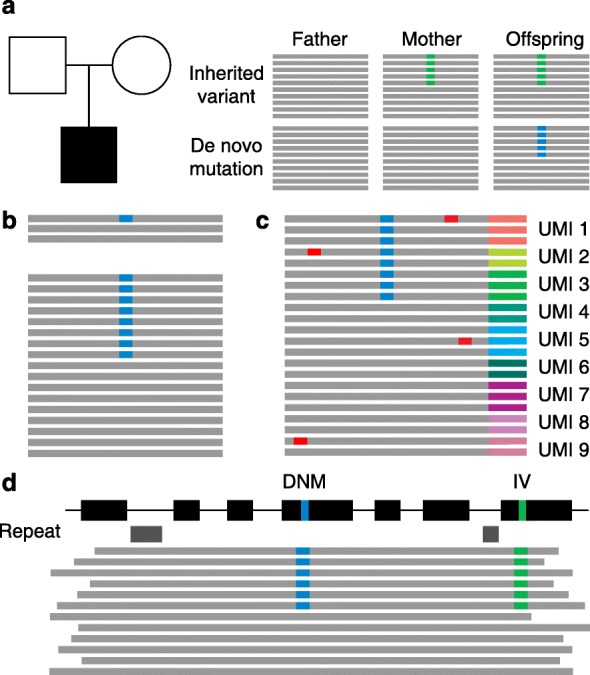
Technical improvements to the detection of de novo mutations (*DNMs*). **a** Trio-based sequencing allows the identification of de novo mutations in an individual. **b** Increased sequencing coverage benefits the detection of de novo mutations (in *blue*). Low coverage (*upper*) reduces the probability that a de novo mutation will be sequenced and called, compared with high sequencing coverage (*lower*). **c** Using random tags or unique molecular identifiers (UMIs) decreases the number of false positives (in *red*) by making consensus calls from all reads with the same UMI. Furthermore, UMIs can be used to remove PCR-derived duplicate reads to determine accurately the allelic ratio. **d** Long sequencing reads improve mappability, even across difficult genomic regions such as those containing repeats (*gray boxes*). Additionally, long reads can be used to phase mutations (shown in *blue* and in *green*) and generate haplotypes, to help identify the parent of origin of a mutation. *IV* inherited variant.

The detection of de novo mutations requires high-quality and high-coverage sequencing (Box Fig. 1b) of three samples; de novo mutations need to be detected in the offspring, and the corresponding base pair needs to be called as wild type in both parental samples in a reliable manner. Poor quality and/or low sequencing coverage of any one of the three analyzed samples severely compromises the reliable detection of de novo mutations. Postzygotic de novo mutations represent an additional challenge as mutations are only present in a small percentage of cells and, upon sequencing, can resemble false-positive sequencing artifacts. For this type of mutation, high sequence coverage is even more crucial. Independent validation by Sanger sequencing (only for validation of germline de novo mutations owing to the low sensitivity of the method) and/or targeted NGS approaches remains essential in the case of uncertainty, especially if a de novo mutation might be of diagnostic relevance. Improvements in raw sequencing quality and higher-coverage sequencing will greatly improve the detection of de novo mutations and allow the consistent identification of postzygotic de novo mutations present in small subsets of cells.

A recent improvement for targeted re-sequencing is single-molecule tracing (Box Fig. 1c), which is based on the incorporation of random tags or unique molecular identifiers (UMIs) to each DNA molecule during capture. UMIs can be used to remove duplicates from the sequencing reads, but they can also allow consensus calling of PCR-derived duplicates of the same DNA molecule [202]. This is of particular interest for the study of mosaicism, in which a mutation is present in only a fraction of all analyzed cells, such as postzygotic de novo mutations [88].

In addition, the affordable and widespread use of long sequencing-read technology (Box Fig. 1d) [203] in the coming years is expected to improve greatly the detection and characterization (including precise breakpoint mapping, length measurement, and exact copy-number state) of small indels, repeat-expansions, as well as CNVs and more-complex structural genomic variation such as inversions and chromosomal rearrangements by improving mappability and even allowing for de novo genome assembly [204–206]. For instance, long-read sequencing technologies identify 85% of novel indels and CNVs with an average size close to 500 bp that were missed by other methods [205]. The application of this technology in parent–offspring trios will provide a better insight into the frequency and role of different types of de novo mutations in health and disease. The use of longer sequencing reads is also particularly useful to determine the parental origin of a de novo mutation that requires mutation phasing (that is, deriving haplotypes) by making use of inherited SNVs on the mutant allele. With currently available short-read sequencing technology, phasing can identify the parental origin for ~20% of de novo mutations [13].

## Box 2 De novo copy number variations and other structural variations

Copy-number variations (CNVs) are defined as deletions or duplications affecting 1000 nucleotides or more [207]. Because of their size, CNVs often have a negative effect on fitness and therefore undergo purifying selection. As a result, there are relatively few inherited CNVs per genome (approximately 160) [4], and de novo germline CNVs are a well-known cause of severe congenital malformations and neurodevelopmental disorders [208–210].

Genomic microarrays have been instrumental for the initial detection and characterization of CNVs with a size below light-microscope resolution [25]. However, NGS has recently shown superior resolution for the detection of CNVs using both short and long sequencing-read approaches and data analysis focusing on depth of sequence reads, split reads, and paired-end reads [211, 212]. The rate at which large de novo CNVs (over 100,000 bp) arise in the human genome is estimated to lie between 0.01 and 0.02 events per generation [25–27]. The mutation rate for indels and CNVs, ranging in size between 10 and 10,000 bp, remains uncertain owing to technical limitations in the detection of these events with short-read sequencing technology.

The CNV mutation rate varies several orders of magnitude depending on the genomic region and parent-of-origin as a result of differences in the mechanism by which the de novo CNV arises [213]. Similar to what has been observed for SNVs, non-recurrent de novo CNVs also show a strong paternal bias and age effect [15, 214]. This correlation highlights a possible mitotic origin for these mutations, resulting from fork stalling and template switching during DNA replication [215]. By contrast, the occurrence of recurrent de novo CNVs, many of which cause well-known developmental syndromes [216], is strongly dependent on the underlying genomic architecture [36]. For instance, the distribution and orientation of segmental duplications (also termed “low-copy repeats”) is known to create “hot spots for structural variation” mediating recurrent CNVs by non-allelic homologous recombination during meiosis (NAHR; Fig. 1) [36, 216]. A strong maternal bias for these types of CNVs has been observed at specific loci [217], which might be explained by a higher local maternal recombination rate. Additionally, for a number of recurrent de novo CNVs, it has been shown that the parental allele carries an inversion that places the duplicated flanking regions in tandem. Some of these inversions have reached high frequencies in specific populations; for instance, the inversion leading to the so-called H2 haplotype on 17q21 is present in 20% of Europeans [218] and predisposes to the occurrence of 17q21 microdeletion syndrome [219].

## Box 3 Establishing causality for a de novo mutation

Although identifying de novo mutations is becoming increasingly easy, interpreting them (i.e., linking them to a phenotype) often remains challenging [220]. Clinical interpretation of de novo mutations requires evaluation at the level of the affected locus or gene, as well as at the variant level [221].

For the interpretation of candidate disease-causing de novo mutations, it is important to verify that the phenotype of the patient with the identified mutation matches that of patients described in the literature possessing similar mutations. Next, the de novo mutation can be evaluated by the same methods used to interpret inherited variations, such as in silico prediction programs such as SIFT, PolyPhen, MutationTaster, and CADD [221–224]. Traditionally, evidence linking a gene or a mutation to a phenotype has been established experimentally [221, 223], although functional validation is laborious and the necessary assays can differ per gene and per mutation. Many recent developments can support the interpretation of de novo mutations in human disease. For instance, to study the consequences of a mutation, induced pluripotent stem cells from patient-derived samples can be differentiated into cell types relevant for the respective disease [225]. Furthermore, as a robust method for in vitro and in vivo genetic manipulation, the “clustered regularly interspaced short palindromic repeats” CRISPR–Cas9 system can be used to establish cell and animal models for functional studies [226, 227]. Other CRISPR/Cas9-based methods, such as “saturation genome editing”, hold promise for the evaluation of hundreds of mutations in a single assay [228], allowing the interpretation of de novo mutations to keep pace with their discovery in the genomics era.

Replication is essential to establish the link between de novo mutations in a novel disease gene and a phenotype [189]. This initially involves the identification of de novo mutations in the same gene in two or more individuals sharing a similar phenotype. However, large-scale parent–offspring sequencing studies have made apparent that this, by itself, is not sufficient to establish causality for a disease [221]. The number of de novo events identified in a specific gene in individuals with the same phenotype must exceed the expected background rate of de novo mutations, which depends on specific features of each gene, such as its size, sequence, and constraint [126]. This approach has been used successfully to identify new disease genes for autism spectrum disorders [229], epileptic encephalopathies [31], and intellectual disability [128]. A novel way to find more patients with de novo mutations in the same gene is emerging from genetic matchmaking platforms such as Matchmaker exchange (http://www.matchmakerexchange.org/) [230] or GeneMatcher (https://www.genematcher.org/) [231], which enable easy data sharing. Establishing unequivocally a link between a genotype and a phenotype requires the same meticulousness in patient phenotyping as in their genotyping, and objective criteria are needed to be able to compare clinical features in patients. For large heterogeneous patient cohorts, systematic phenotyping, including an assessment in Human Phenotype Ontology (HPO) terms, can prove beneficial and increase the diagnostic yield in exome sequencing [127, 232–234].

Large-scale databases of genetic variation can be used to see whether a gene or gene region shows constraint against variation in controls, as the frequency of a mutation in the population is often a good indirect estimation of its pathogenicity [223]. To this end, RVIS and selective constraint scores have become routine in the interpretation of de novo variants, both in research and in the clinic [126, 188]. Population databases, such as the Exome Aggregation Consortium (ExAC) [55], are expected to be depleted of de novo disease-causing mutations for severe and early-onset disorders. Given that de novo mutations are the rarest type of variation, the absence of a mutation from the ExAC database is not in itself sufficient evidence for its pathogenicity. By contrast, the presence of a mutation in ExAC does not automatically entail that the mutation is not disease causing. Pathogenic mutations involved in dominant disease are present in ExAC [55], which might be explained by variable penetrance for these variants [235], the presence of false-positive variants in the control database [236], or undiagnosed disease in control individuals. Possible other explanations for these observations could be the presence of these mutations as somatic events in control individuals [106–108] or resilience to disease in a few selected individuals [237].

## Abbreviations

CHD: Congenital heart defect
CNV: Copy number variation
DNM: De novo mutation
ExAC: Exome Aggregation Consortium
Indel: Insertion–deletion
MMR: Mismatch repair
NAHR: Non-allelic homologous recombination
NGS: Next-generation sequencing
PAE: Paternal age effect
PGC: Primordial germ cell
rDNA: Ribosomal DNA
RVIS: Residual variation intolerance score
SNV: Single-nucleotide variant
UMI: Unique molecular identifier
WES: Whole-exome sequencing
WGS: Whole-genome sequencing
